# Serum Uric Acid Revealed a U-Shaped Relationship With All-Cause Mortality and Cardiovascular Mortality in High Atherosclerosis Risk Patients: The ASSURE Study

**DOI:** 10.3389/fcvm.2021.641513

**Published:** 2021-05-24

**Authors:** Yan Cang, Shaojie Xu, Jingyin Zhang, Jingyi Ju, Zijun Chen, Keke Wang, Jue Li, Yawei Xu

**Affiliations:** ^1^Department of Cardiology, Shanghai Tenth People's Hospital, Tongji University School of Medicine, Shanghai, China; ^2^Tongji University School of Medicine, Shanghai, China; ^3^Department of Cardiology, Clinical Medical College of Shanghai Tenth People's Hospital, Medical Department of Soochow University, Suzhou, China

**Keywords:** serum uric acid, all-cause mortality, cardiovascular mortality, framingham risks, atherosclerosis risks

## Abstract

**Background:** Previous studies have demonstrated an association between hyperuricemia and cardiovascular disease (CVD). The Framingham study confirmed that patients with high atherosclerotic risks (HARs) had worse prognoses. However, after adjusting for confounding factors, the association between serum uric acid (SUA) and all-cause mortality and cardiovascular mortality remains unclear, especially for HAR patients.

**Objective:** The aim of this study was to reveal the relationship of SUA with all-cause and cardiovascular mortality in HAR patients.

**Methods:** This multicenter cohort study enrolled 3,047 participants, and the follow-up was 68.85 ± 11.37 months. Factors related to cardiovascular and all-cause mortality were tested by multivariate Cox regression analysis. Restricted cubic splines (RCSs) with knots were used to explore the shape of the dose–response relationship with SUA and the hazard ratio (HR) of all-cause and CVD mortality. SUA transformed by RCS was added to the Cox regression model as an independent variable, and all-cause and CVD mortality scores were calculated. Survival receiver operating characteristic curves were produced using a regression model predicting the score.

**Results:** SUA demonstrated a “U-shaped” relationship with all-cause and cardiovascular mortality. SUA predicted all-cause and CVD mortality, with cutoff values of values of >370.5 μmol/L for males and >327.65 μmol/L for females and <180.5 μmol/L for males and <165.7 μmol/L for females, respectively. The survival ROC curve indicated that SUA is able to predict all-cause and CVD mortality, with areas under the curve of 0.702 and 0.711, respectively. The HRs of all-cause mortality (male and female) with hyperuricemia and hypouricemia were 2.08 and 2.01 and 2.04 and 1.98, respectively, and the HRs of CVD mortality (male and female) were 2.09 and 1.79, and 2.02 and 1.89, respectively.

**Conclusion:** Abnormal SUA levels were significant and independent risk factors for all-cause and CVD mortality. Hyperuricemia and hypouricemia increased mortality in both males and females. Routine SUA evaluation and intensive management are needed for HAR patients.

**Clinical Trial Registration:** www.ClinicalTrials.gov, identifier: NCT03616769.

## Background

Previous studies in humans have demonstrated an association between hyperuricemia, arterial stiffness, and endothelial dysfunction (1), and the serum uric acid (SUA) level has been suggested to be an important modulator of the inflammatory process (2). Several studies have reported that SUA serves as a marker of an underlying pathophysiological process (3, 4), and some evidence shows that elevated SUA concentrations are associated with a higher risk of hypertension and cardiovascular disease (CVD) (5–7). However, the relationship between SUA level and CVD mortality remains controversial because established cardiovascular risk factors are complex and may be considered a confounding factor (8). Although previous studies have indicated that hyperuricemia plays a role in atherogenesis in the development of CVD (9–11), there is little research available on the relationship between hypouricemia and hypouricemia with all-cause and CVD mortality.

In addition, the Framingham Heart Study found that patients with high atherosclerotic risk (HAR) have worse CVD prognosis (12–14). However, the synergistic effect of SUA combined with HAR on prognosis is still unclear. Although a few multicenter cohort studies have also focused on abnormal SUA levels combined with HAR, most studies were from Western countries and lack Asian data, especially in Chinese patients. Therefore, the aim of this research was to explore relationships among SUA levels, all-cause mortality, and cardiovascular mortality in HAR patients.

## Methods

### Study Population

**Figure 1:**
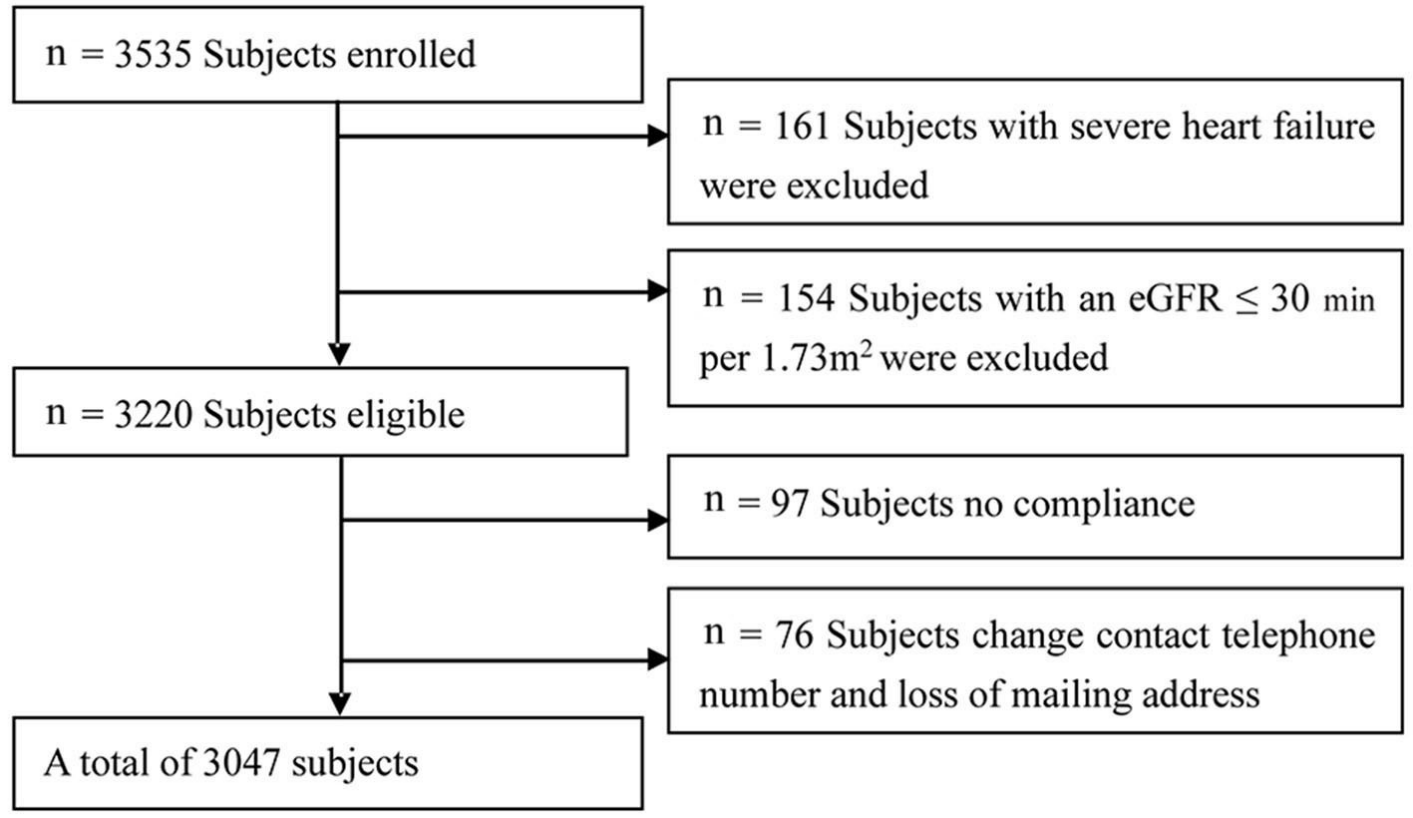
Study flowchart.

The ASSURE study (ClinicalTrials.gov identifier NCT03616769) is a multicenter prospective cohort study. The first cross-sectional survey was conducted in 2011. Eligible participants were followed up from November 2011 to June 2018 (mean follow-up was 68.71 ± 11.35 months). During the follow-up period, 76 subjects had missing data, and 97 were not compliant. Thus, the study sample comprised 3,047 eligible participants (1,625 male and 1,422 female participants) older than or equal to 35 years (mean age 60.2 ± 10.4 years). A total of hospitalized subjects were consecutively enrolled from the Cardiology Department of Beijing University Affiliated and of Shanghai Tongji University–affiliated hospitals. All subjects were under treatment for CVD. The inclusion criterion was HAR. The exclusion criteria were severe congestive heart failure and severe renal failure. Severe congestive heart failure was defined as greater than or equal to cardiac functional classification 3 formulated by the New York Heart Association (NYHA). Severe renal failure was defined as an estimated glomerular filtration rate (eGFR) <30 mL/min per 1.73 m^2^ (Figure 1). All participants provided written informed consent for this study, which was approved by the Ethics Committee of Tongji University.

### Cardiovascular Event Definitions

Hospitalized myocardial infarction (MI) was classified as definite or probable based on chest pain symptoms, cardiac enzyme levels, electrocardiographic (ECG) findings, or angioplasty. Coronary heart disease (CHD) was determined to be present if there was (1) ECG evidence of a prior MI, (2) prior coronary artery bypass surgery or angioplasty, (3) coronary angiography showing CHD, (4) symptoms of angina and ECG revealing myocardial ischemia performance or laboratory tests showing increased cardiac enzymes and excluding other types of disease, or (5) a self-reported history of a physician-diagnosed heart attack. Coronary artery disease (CAD) death was classified as “definite” based on chest pain symptoms, hospital records, and medical history.

### Assessment of Cardiovascular Events and Identification of Death From All Causes and CVD

Cardiovascular events are composed of cardiac events, including non-fatal MI, unstable angina, and coronary revascularization procedures, during the follow-up period. The exclusion criteria were stable angina (>6 months), revascularization procedure for CAD (>6 months), and MI (>6 months).

In this study, cardiovascular death was the only cardiac event death considered. Medical records and death certificates for all patients who had an event were obtained and validated by a cardiologist. Death was confirmed from hospital records or by contact with participants and their families. All materials were reviewed independently by five senior physicians of the cohort study to confirm the cause of death.

### Hyperuricemia and Hypouricemia Definitions

Hyperuricemia refers to consuming a normal-purpurin diet, with two determinations on different days: SUA ≥420 μmol/L or 7 mg/dL (male), ≥ 357 μmol/L or 6 mg/dL (female) (15). Hypouricemia refers to consuming a normal-purpurin diet, with two determinations on different days: SUA ≤178 μmol/L or ≤3 mg/dL (male) and SUA ≤149 μmol/L or ≤2.5 mg/dL (female) (16, 17).

### Framingham Risk Score and High Atherosclerosis Risk

The Framingham risk score (FRS) was calculated based on coronary risk factors, including age, sex, total cholesterol (TC), low-density lipoprotein cholesterol (LDL-C), hypertension, and smoking status, according to the National Cholesterol Education Program Adult Treatment Panel III algorithm (18). The calculated total scores were used to estimate the 10-year CHD risk in participants without previous CVD and when an FRS > 20% or between 10 and 20% was considered HAR (18).

### Framingham Risk Factors

The diagnostic criteria for hypertension were as follows: patients taking antihypertensive medication or systolic blood pressure (SBP) ≥140 mm Hg or diastolic blood pressure ≥90 mm Hg (1 mm Hg = 133 kPa) or both. The criterion for hypertension was a single hypertensive disease.

Similarly, the criterion for dyslipidemia was a single dyslipidemic disease. The definition of dyslipidemia is abnormalities in serum levels of lipids, including overproduction or deficiency. Abnormal serum lipid profiles include high TC, high triglycerides (TGs), low HDL-C, and elevated LDL-C.

### Measurement of Ankle and Arm Blood Pressures

Qualified ultrasonographers measured ankle and brachial SBPs and Doppler ultrasound (Nicolet Vascular, Elite 100R, USA) was used to measure SBP in the bilateral brachial, tibial, and dorsal pedal arteries. The ankle–brachial index (ABI) has been shown to be a powerful independent marker of cardiovascular risk, with predictive ability similar to that of the Framingham criteria (19, 20).

A questionnaire was designed to collect from all participants information about general characteristics, diagnosis, medical history and related factors, medical treatment, and biochemical examination.

### Baseline Measurements

Factors were assessed for all subjects, including daily habits, medical histories, and blood samples. These blood samples were used to measure TC, TGs, HDL-C, LDL-C, serum creatinine (Cr), SUA, glucose, and fasting plasma glucose. Blood samples were drawn from an antecubital vein into a Vacutainer using a 19-gauge needle, and serum concentrations were assessed with commercially available kits. The GFR was calculated as GFR (mL/min per 1.73 m^2^) = 186 × creatinine^−1.154^ × age^−0.203^ and GFR (mL/min per 1.73 m^2^) = 142 × creatinine^−1.154^ × age^−0.203^ for males and females, respectively. Glucose was measured using a Hitachi 717 analyzer (Roche) with enzymatic reagents from Roche. The presence of symptomatic peripheral arterial disease (PAD) was evaluated by the Rose questionnaire (21). A previous MI or ischemic stroke was documented by hospital records. Physical examination data included body mass index (BMI), blood pressure, and ABI. Severe congestive heart failure was defined as greater than or equal to cardiac functional classification 3 formulated by the NYHA. Severe renal failure was defined as an eGFR < 30 mL/min per 1.73 m^2^.

### Follow-Up Methods

For follow-up, participants were contacted by physicians of the cohort study at annual intervals. Outcomes were obtained based on the annual phone interviews, 6-year follow-up examinations, hospital records, and death records. The primary clinical event endpoints of this study were estimated all-cause mortality and cardiovascular mortality. The secondary endpoints were CHD and CHD risk equivalent, including PAD, stroke, diabetes mellitus (DM), and CVD. Follow-up time was the number of years from the baseline (first) visit. For subjects who had more than one event, all clinical events were considered in the analysis.

### Statistical Analysis

All analyses were performed using the R statistical package (version 3.6.2) [http://www.r-project.org (22)]. Continuous variables are expressed as the mean ± SD and categorical variables as percentages. Continuous and categorical variable difference comparisons were made by independent-samples ANOVA (analysis of variance) and the χ^2^ test, as appropriate. The Kruskal–Wallis test was used to evaluate non-normally distributed continuous variables. A *P* < 0.05 was considered statistically significant. Due to a skewed distribution, TC, TG, HDL-C, LDL-C, Cr, and SUA were logarithm-transformed (log) for analyses. Crude death from all-cause and CVD was assessed by SUA stratification. Cumulative event rates were estimated with Kaplan-Meier survival curves, and probability values were calculated with the log-rank test. Cox proportional hazard analyses were performed to test the association of SUA and death from all causes or CVD. A Cox regression model was adjusted for potential confounders, including age, sex, duration of hypertension, smoking status, dyslipidemia history, chronic renal insufficiency history, DM history, percutaneous coronary angioplasty (PTCA) history, coronary artery bypass grafting (CABG) history, PAD history, MI history, ischemic stroke history, hypertension, ABI, FRS, eGFR, diuretics, center, and year of screening examination. Potential confounding variables with *P* < 0.10 were adjusted for multivariate analysis. Restricted cubic splines (RCSs) with knots were used to further explore the shape of the dose–response relationship between SUA level and the hazard ratio (HR) of all-cause mortality and CVD mortality. Knots were at the 5th, 50th, 90th, and quartile of the SUA distribution. Missing values were handled by K-means clustering imputation. All *P*-values were 2-tailed, and a value < 0.05 was considered significant. The optimized prognostic cutoff value of SUA was analyzed with X-Tile Software (version 3.6.1, https://medicine.yale.edu/lab/rimm/research/software). SUA transformed by RCS was added to the Cox regression mode as an independent variable, and all-cause CVD mortality scores were calculated. A survival receiver operating characteristic (ROC) curve was used to determine the predictive effect of SUA level and its variability on all-cause mortality and CVD mortality. The ROC curve and area under the curve (AUC) were implemented with a regression model predicting the score.

## Results

### Baseline Characteristics

A total of 3,220 eligible participants with available baseline data were enrolled. Among the participants, 76 individuals had missing follow-up data because of changing the telephone number or family address during follow-up; 97 subjects were not compliant (Figure 1). Therefore, the study sample actually comprised 3,047 participants. Based on careful calculation, the missing participants did not significantly affect the results. Our research showed hyperuricemia and hypouricemia prevalences of 18.1 and 16.7%, respectively, and the average SUA for the entire cohort was 322.65 ± 33.12 μmol/L (standard deviation). According to SUA level, values were subdivided into 0–242.0, 242.0–312.0, 312.0–386.75, and more than 386.75 μmol/L subgroups. As shown in Table 1, among all variables examined, abnormal SUA was associated with hypertension, a higher proportion of male subjects, older age, a higher level of Cr, diuretic use, and chronic renal insufficiency. Regarding subjects with HAR, there was no significant difference for ischemic stroke, MI history, CABG history, PTCA history, DM, dyslipidemia, and smoking. Of note, significant differences were observed for female participants with FRS risk stratification. However, FRS risk stratification by subgroup was not significant in male participants. In contrast with the reference group, the participants were more likely to be treated with ACEI.

**Table 1:** Comparison of subjects' baseline characteristics of sex categories according to serum uric acid level.

	**Male**					**Female**				
**Characteristics**	**Quartile of SUA levels**, **μmol/L**				***P*-value**	**Quartile of SUA levels**, **μmol/L**				***P*-value**
	**0–242.0**	**242.1–312.0**	**312.1–386.75**	**>386.75**		**0–242.0**	**242.1–312.0**	**312.1–386.75**	**>386.75**	
Age (years)	67.3 ± 11.3	65.8 ± 11.7	65.7 ± 11.8	68.0 ± 11.5	0.002	65.6 ± 10.8	67.3 ± 10.5	67.7 ± 9.7	70.4 ± 9.9	< 0.001
Diabetes, *n* (%)	198 (41.8)	174 (35.9)	154 (31.8)	159 (32.9)	0.006	310 (43.7)	189 (42.3)	134 (45.1)	99 (38.2)	0.372
DM duration (years)	2.8 ± 5.0	3.0 ± 6.0	2.3 ± 4.8	2.5 ± 5.0	0.173	3.6 ± 6.1	3.8 ± 6.4	3.8 ± 7.1	3.6 ± 6.9	0.925
Hypertension, *n* (%)	309 (65.2)	324 (66.8)	343 (70.7)	380 (78.7)	< 0.001	479 (78.7)	347 (78.7)	252 (84.8)	216 (83.4)	< 0.001
HT duration (years)	8.5 ± 11.5	8.9 ± 11.2	9.7 ± 12.2	10.8 ± 11.3	0.012	8.3 ± 10.8	10.5 ± 11.6	12.1 ± 11.8	12.6 ± 13.1	< 0.001
Dyslipidemia, *n* (%)	134 (34.6)	152 (38.0)	185 (46.7)	171 (40.8)	0.005	271 (46.5)	161 (42.5)	117 (50.0)	98 (44.1)	0.297
DL duration (years)	1.3 ± 0.31	1.8 ± 0.45	1.8 ± 0.41	2.1 ± 0.46	0.069	1.9 ± 0.39	1.9 ± 0.43	2.0 ± 0.42	1.6 ± 0.34	0.532
Smoking, *n* (%)	310 (65.4)	307 (63.3)	325 (67.0)	320 (66.3)	0.648	57 (8.0)	48 (10.7)	27 (9.1)	29 (11.2)	0.318
Smoking duration (years)	20.7 ± 1.86	20.0 ± 1.81	21.4 ± 1.85	21.3 ± 1.91	0.552	2.7 ± 1.02	3.4 ± 1.08	2.7 ± 0.97	4.3 ± 1.32	0.218
MI history, *n* (%)	87 (18.4)	83 (17.1)	94 (19.4)	102 (21.1)	0.439	60 (8.5)	51 (11.4)	34 (11.4)	27 (10.4)	0.307
Diuretics, *n* (%)	105 (22.2)	100 (20.7)	132 (27.2)	195 (40.5)	< 0.001	140 (19.8)	116 (26.0)	106 (35.8)	111 (43.0)	< 0.001
PTCA history, *n* (%)	56 (11.8)	70 (14.4)	69 (14.2)	64 (13.3)	0.631	59 (8.3)	42 (9.4)	24 (8.1)	18 (6.9)	0.721
CABG history, *n* (%)	18 (3.8)	16 (3.3)	19 (3.9)	20 (4.1)	0.918	9 (1.3)	7 (1.6)	7 (2.4)	5 (1.9)	0.634
IS history, *n* (%)	182 (38.4)	163 (33.6)	161 (33.2)	167 (34.6)	0.315	212 (29.9)	139 (31.1)	83 (27.9)	83 (32.0)	0.719
CRI history, *n* (%)	31 (6.8)	29 (6.3)	38 (8.1)	83 (17.6)	< 0.001	56 (8.1)	29 (6.6)	32 (11.0)	53 (21.1)	< 0.001
TG, mmol/L	1.4 ± 0.80	1.5 ± 0.90	1.6 ± 1.00	1.8 ± 1.50	< 0.001	1.6 ± 1.10	1.8 ± 1.3 0	2.0 ± 1.40	2.0 ± 1.20	< 0.001
HDL-c, mmol/L	1.2 ± 0.40	1.2 ± 0.3 0	1.1 ± 0.40	1.1 ± 0.50	0.106	1.3 ± 0.40	1.2 ± 0.30	1.3 ± 0.40	1.2 ± 0.40	0.001
LDL-c, mmol/L	2.5 ± 0.80	2.7 ± 0.80	2.7 ± 0.90	2.6 ± 0.80	< 0.001	2.8 ± 0.90	2.9 ± 0.80	3.0 ± 0.90	2.8 ± 1.00	0.052
CRE	93.6 ± 7.39	103.2 ± 8.06	109.2 ± 8.55	138.2 ± 13.17	< 0.001	79.0 ± 6.73	84.9 ± 6.83	91.2 ± 6.53	143.0 ± 15.90	< 0.001
Blood glucose	6.8 ± 0.30	6.4 ± 0.27	6.0 ± 0.24	6.1 ± 0.26	< 0.001	6.7 ± 0.32	6.6 ± 0.28	6.6 ± 0.28	6.6 ± 0.32	0.816
BMI, kg/m^2^	23.2 ± 3.4	24.7 ± 3.3	24.6 ± 3.4	24.9 ± 3.7	< 0.001	23.8 ± 3.6	24.3 ± 3.8	25.1 ± 3.5	24.9 ± 4.0	< 0.001
ABI	1.01 ± 0.20	1.02 ± 0.20	0.99 ± 0.20	0.99 ± 0.20	0.066	0.99 ± 0.20	0.97 ± 0.20	0.95 ± 0.20	0.91 ± 0.30	< 0.001
FRS^†^, *n* (%)	454 (24.7)	459 (25.5)	461 (25.3)	462 (25.1)	0.910	408 (21.9)	446 (24.8)	296 (16.7)	258 (13.6)	< 0.001
FRS^††^, *n* (%)	357 (24.1)	379 (25.5)	375 (25.3)	373 (25.1)	0.647	488 (27.2)	424 (23.1)	287 (15.6)	247 (12.4)	< 0.001

*SBP, Systolic blood pressure; DBP, diastolic blood pressure; HT, hypertension; BMI, body mass index; TC, total cholesterol; TG, triglycerides; FPG, fasting plasma glucose; HDL-c, high-density lipoprotein; LDL-c, low-density lipoprotein; Cr, serum creatinine; SUA, serum uric acid; DM, diabetes mellitus; MI, myocardial infarction; IS, ischemic stroke; CRI, chronic renal insufficiency; DL, dyslipidemia; CVD, cardiovascular disease; PAD, peripheral arterial disease; ABI, ankle–brachial index; PTCA, percutaneous coronary angioplasty; HR, hazard ratio; CABG, coronary artery bypass grafting; FRS, Framingham risk score.*

† *FRS analysis of participants with FRS 10–20%.*

†† *FRS analysis of participants with FRS; >20% was identified as high risk for 10-year coronary heart disease*.

### All-Cause and CVD Mortality Rates in SUA Quartile Groups

After multivariable adjustment, Cox regression models revealed that compared with the SUA (242.0–312.0) subgroup, all-cause mortality was 24.5, 19.1, 22.9, and 35.9% in the SUA (0–242.0), SUA (312.0–386.75), and SUA >386.75 μmol/L subgroups, respectively (Table 2); CVD mortality rates were 12.2, 10.8, 13.4 and 18.3%, respectively. HRs of all-cause mortality and cardiovascular mortality were 2.05 (95% CI = 1.35–2.90), 1.85 (95% CI = 1.54–2.76), and 2.11 (95% CI = 1.59–3.07) and 1.95 (95% CI = 1.29–2.90), 1.70 (95% CI = 1.05–2.81), and 2.42(95% CI = 1.61–3.12), respectively, for the above subgroups.

**Table 2:** Adjusted hazard ratio for all-cause mortality and cardiovascular disease (CVD) mortality by Cox regression models according to serum uric acid level.

**Variable**	**SUA**				***P* for difference**
**Characteristic SUA level, μmol/L**	**Quartile 1**	**Quartile 2**	**Quartile 3**	**Quartile 4**	
	**0–242.0**	**242.0 < SUA ≤ 312.0**	**312.0 < SUA ≤ 386.75**	**386.75 < SUA**	
All-cause mortality	973	789	672	613	
No. of deaths	238 (24.5%)	151 (19.1%)	154 (22.9%)	220 (35.9%)	
Multivariable adjustment					
Model 1	2.13 (1.45–3.09)	1	1.94 (1.23–2.92)	2.20 (1.73–3.17)	< 0.001
Model 2	2.10 (1.21–3.12)	1	1.90 (1.19–2.98)	2.17 (1.70–3.09)	< 0.001
Model 3	2.06 (1.35–2.90)	1	1.86 (1.54–2.89)	2.12 (1.63–3.17)	< 0.001
Model 4	2.05 (1.35–2.90)	1	1.85 (1.54–2.76)	2.11 (1.59–3.07)	< 0.001
CV mortality	973	789	672	613	
No. of deaths	119 (12.2%)	85 (10.8%)	90 (13.4%)	112 (18.3%)	
Multivariable adjustment					
Model 1	2.21 (1.32–3.04)	1	1.82 (1.36–2.94)	2.57 (1.77–3.32)	< 0.001
Model 2	2.18 (1.43–2.97)	1	1.81 (1.32–2.93)	2.53 (1.71–3.30)	< 0.001
Model 3	1.98 (1.32–2.94)	1	1.71 (1.08–2.85)	2.45 (1.67–3.22)	< 0.001
Model 4	1.95 (1.29–2.90)	1	1.70 (1.05–2.81)	2.42 (1.61–3.12)	< 0.001

*Model 1 was adjusted for age and sex, estimated glomerular filtration rate (eGFR), and diuretic use. Model 2 was adjusted for model 1 covariates and smoking, alcohol drinking, use of diuretics, a history of heart failure, a history of diabetes, a history of renal insufficiency, a history of metabolic syndrome, and a history of stroke. Model 3 was adjusted for model 2 covariates ABI (ankle–brachial index) and FRS (Framingham risk score). Model 4 was adjusted for model 3 covariates central effect, year of screening examination*.

### Survival Analysis of SUA Quartile Groups

**Figure 2:**
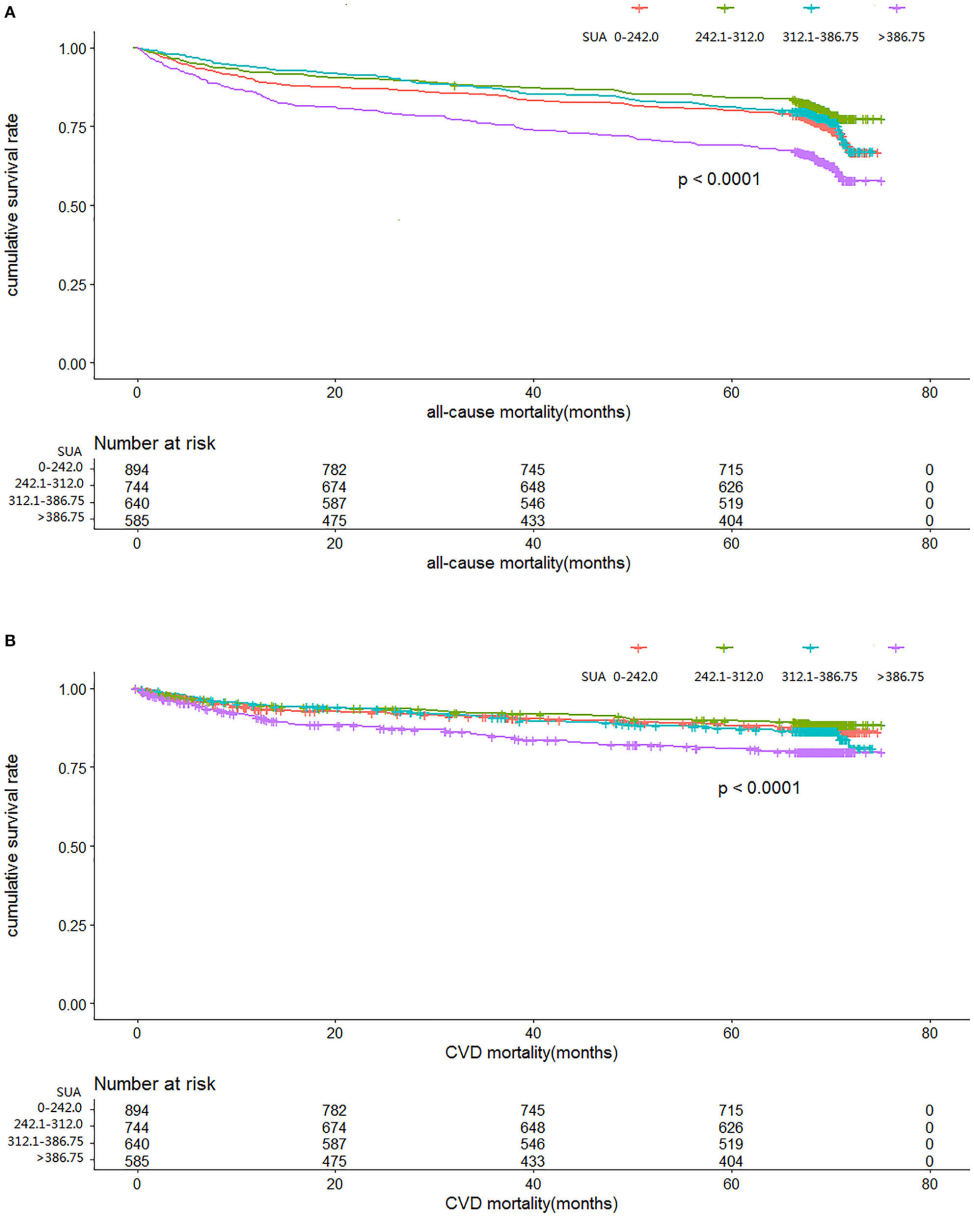
Time course to death from all causes **(A)** and cardiovascular disease (CVD) **(B)** according to serum uric acid level in the cohort study during 6-year follow-up.

**Figure 3:**
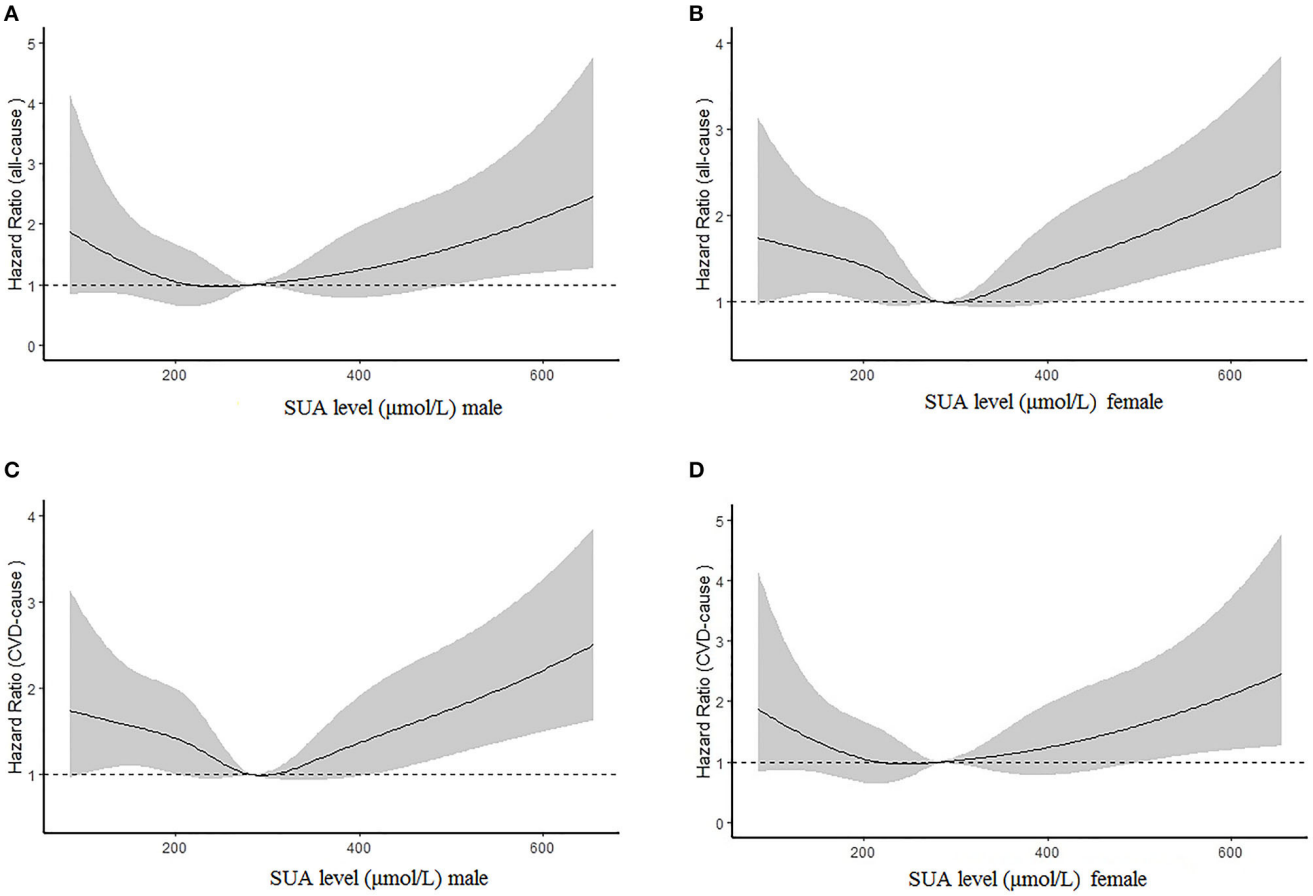
Multivariable adjusted cubic spline models for the association between serum uric acid (SUA) level and hazard ratios (HRs) for all-cause mortality among males **(A)** and females **(B)** and cardiovascular disease (CVD) mortality between males **(C)** and females **(D)**.

Figures 2A,B illustrate the relationship among the SUA quartile categories of all-cause mortality and CVD mortality, respectively. Kaplan–Meier curves of survival showed the highest all-cause mortality and cardiovascular mortality for the SUA >386.75 μmol/L subgroup, followed by the SUA 0–242.0 and 312.0–386.75 μmol/L subgroups. The SUA 242.0–312.0 μmol/L subgroup had the least mortality. As depicted in Figure 3, after multivariable adjustment, cubic spline models showed a U-shaped association between SUA level with all-cause mortality (A, B) and CVD-cause mortality (C, D) among males and females, respectively.

### Survival Analysis of SUA Values According to ROC

**Figure 4:**
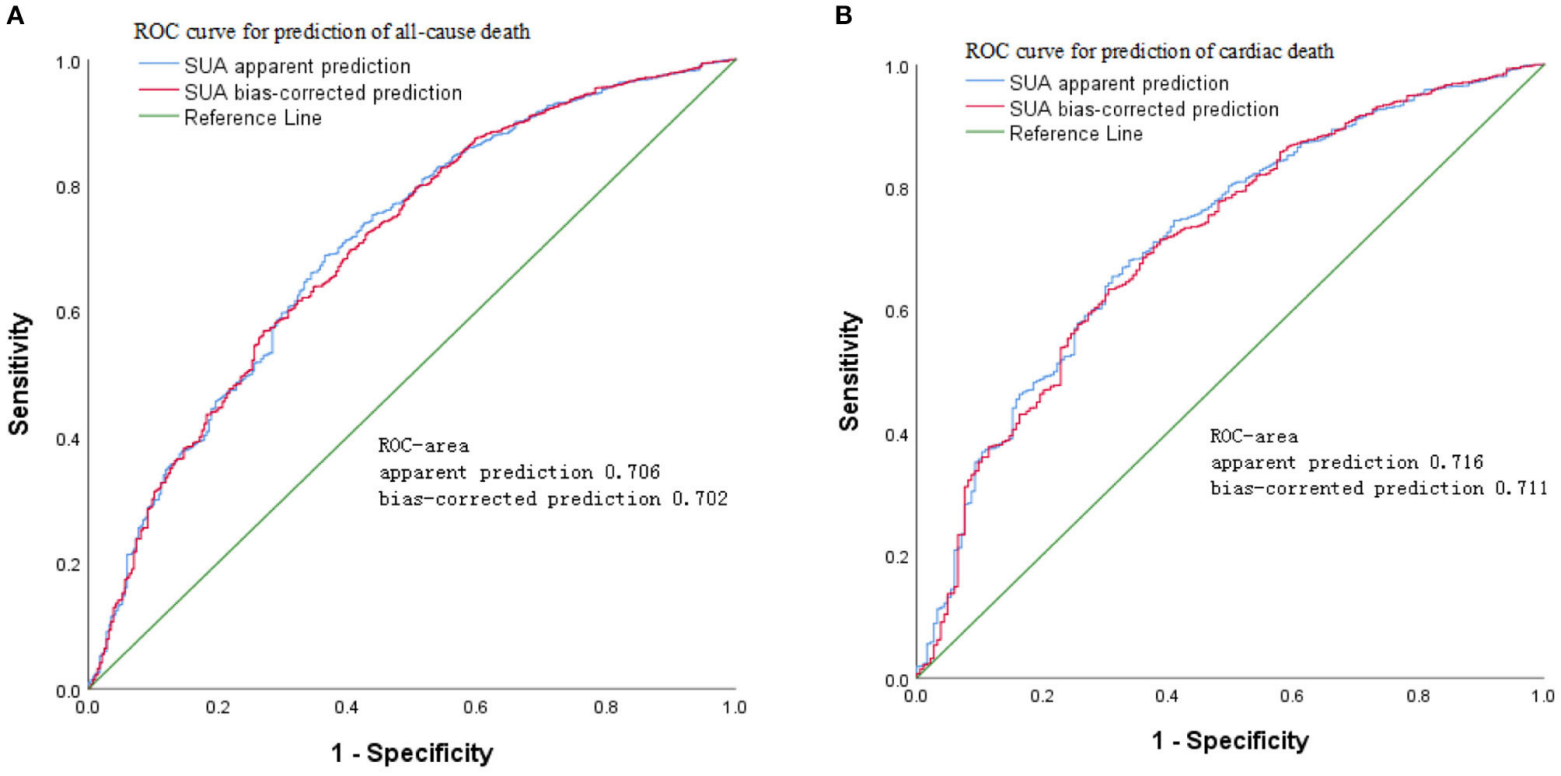
Age- and sex-adjusted receiver operating characteristic (ROC) curves for predicting **(A)** all-cause mortality and **(B)** cardiovascular disease (CVD) mortality according to serum uric acid (SUA) in the cohort study during 6-year follow-up.

The results of age- and sex-adjusted survival ROCs (Figure 4) indicated that SUA level can predict all-cause mortality, with an AUC of apparent prediction of 0.706 (95% CI = 0.696–0.727; *P* < 0.001); when adjusted for age and sex, the AUC was 0.702 (95% CI = 0.692–0.725, *P* < 0.001). Meanwhile, SUA levels predicted CVD-cause mortality, and the AUC of apparent prediction was 0.716 (95% CI = 0.701–0.747, *P* < 0.001); when adjusted for age and sex, the AUC was 0.711 (95% CI = 0.697–0.742, *P* < 0.001).

### HR of All-Cause and Cardiovascular Mortality According to SUA Cutoff Value

**Figure 5:**
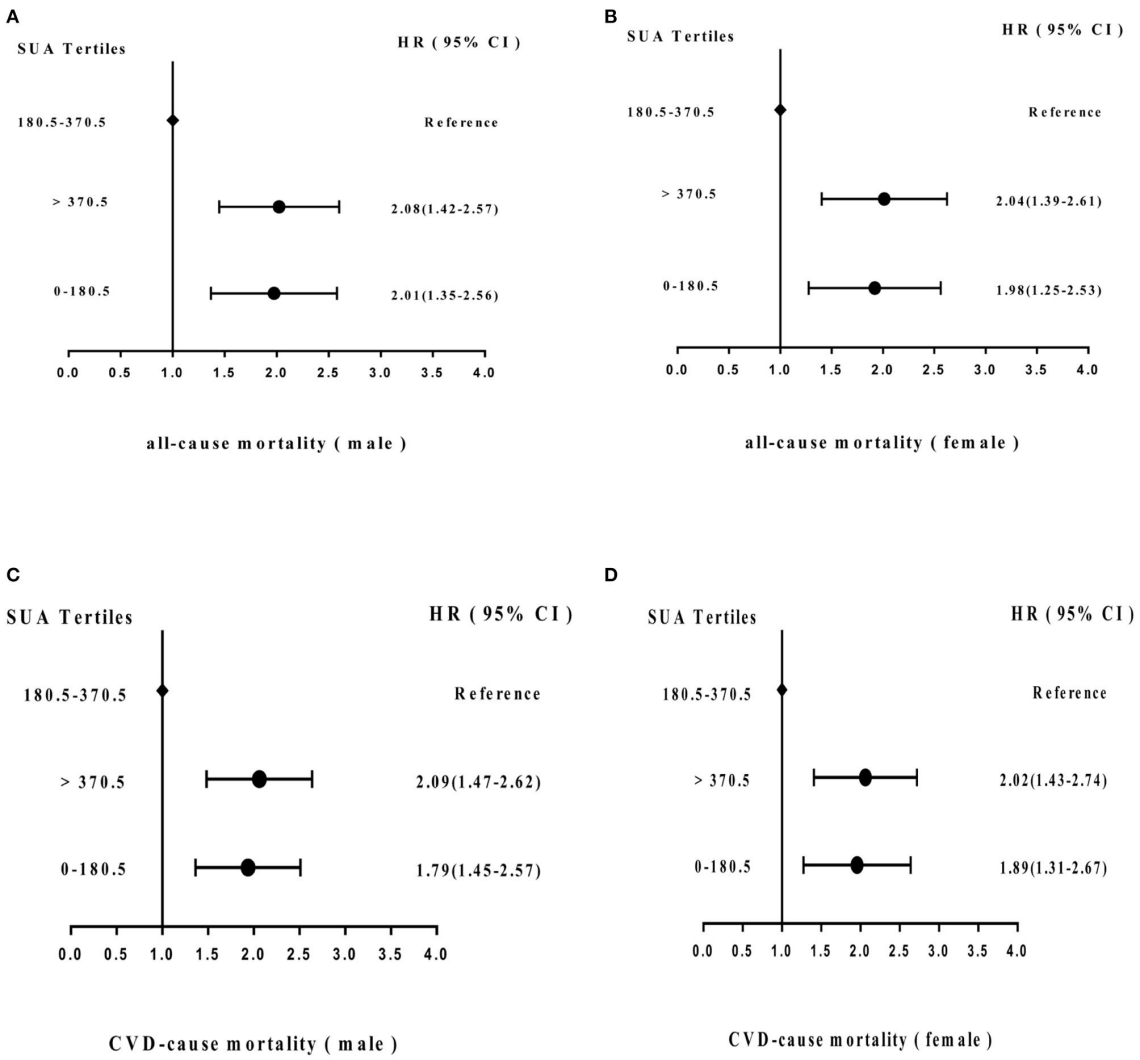
Adjusted hazard ratios (HRs) for **(A)** all-cause mortality (male) and **(B)** (female) and **(C)** cardiovascular disease (CVD) mortality (male) and **(D)** (female) according to cutoff values of SUA level in the cohort study during 6-year follow-up. CI, confidence interval.

Figure 5 shows the adjusted proportional HR of mortality according to SUA cutoff value. Compared with the SUA 180.5–370.5 μmol/L subgroup, HRs for all-cause mortality and cardiovascular mortality in males in the SUA 0–180.5 and SUA >370.5 μmol/L subgroups were 2.01 (95% CI = 1.35–2.56) and 2.08 (95% CI = 1.42–2.57) and 1.79 (95% CI = 1.45–2.57) and 2.09 (95% CI = 1.47–2.62), respectively, after adjusting for age, hypertension, stroke, MI history, smoking status, chronic renal disease, PAD history, PTCA history, and CABG history. In females, HRs of all-cause and CVD mortality were 1.98 (95% CI = 1.25–2.53) and 2.04 (95% CI = 1.39–2.61), and 1.89 (95% CI = 1.31–2.67) and 2.02 (95% CI = 1.43–2.74), respectively.

### Other Mortality Risk

**Figure 6:**
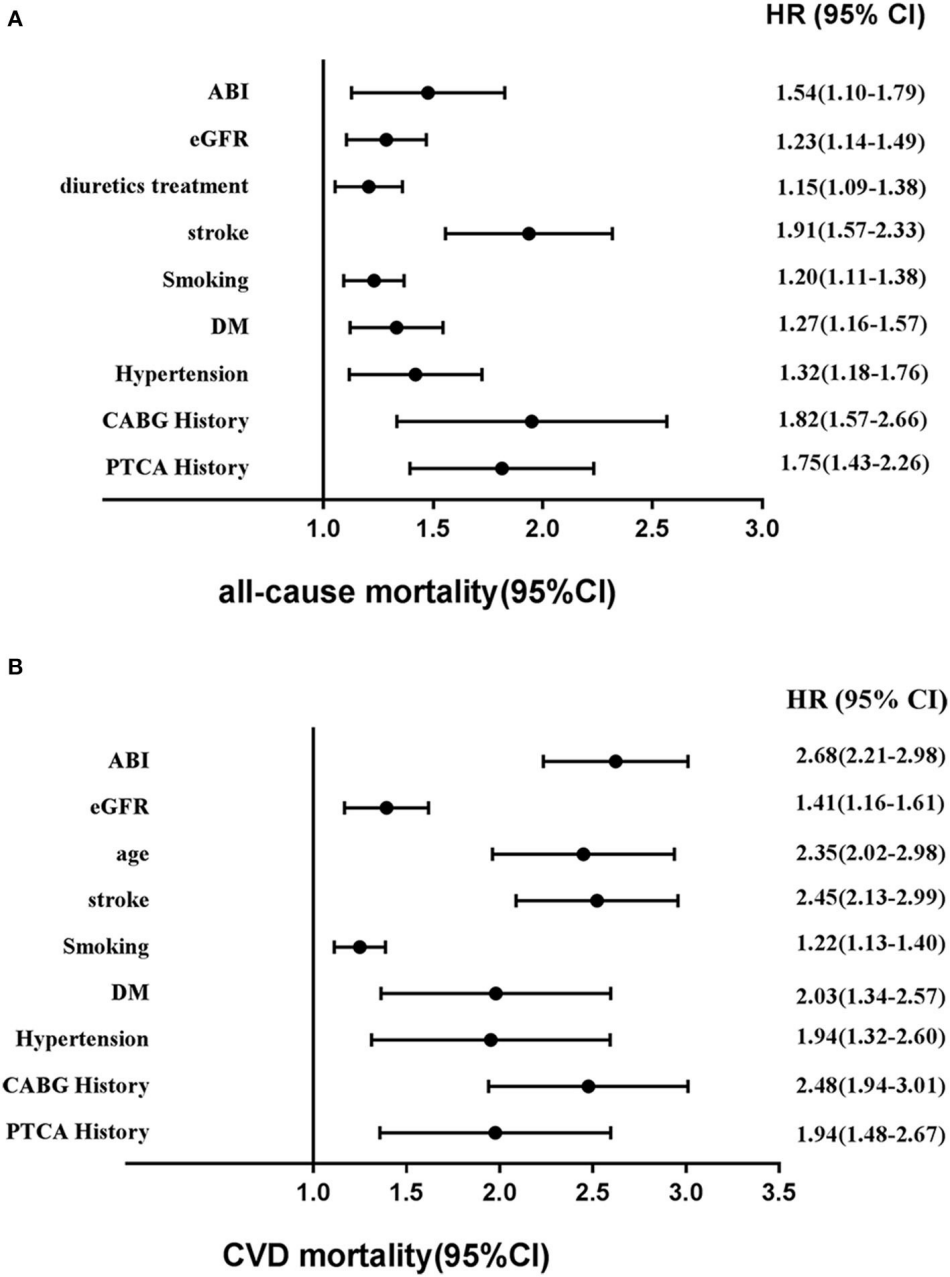
Adjusted other hazards ratios for **(A)** all-cause mortality and **(B)** CVD mortality in the cohort study during 6-year follow-up. CVD, cardiovascular disease; HR, hazards ratio; CI, confidence interval; eGFR, estimated glomerular filtration rate; ABI, ankle**–**brachial index.

Based on Figures 6A,B, Cox regression models also demonstrated that factors for risk of mortality included sex, age, hypertension, stroke, metabolic syndrome, DM, MI history, PAD history, hyperlipidemia history, smoking status, PTCA history, CABG history, and low eGFR. Of note, among these risk factors, age, hypertension, stroke, DM, lower eGFR, abnormal ABI, MI history, and CABG history occupied major positions.

## Discussion

Because of the close relationship between SUA level, dietary structure, and obvious diet differences among northern and southern China, we selected in-hospital patients from Beijing and Shanghai to represent these areas. In this cohort study, after further adjusting for potential confounders, the main findings according to SUA levels suggest a U-shaped, independent relationship, instead of a dose–response relationship, between SUA level and all-cause mortality and CVD mortality in both males and females. Furthermore, the findings indicate that long-term average SUA levels >370.5 μmol/L or 6.22 mg/dL (male) and >327.65 μmol/L or 5.50 mg/dL (female) significantly increase the risk of all-cause mortality and CVD mortality. Moreover, long-term low-level SUA <180.5 μmol/L or 3.03 mg/dL (male) <165.7 μmol/L or 2.78 mg/dL (female) was an independent risk factor for all-cause mortality and CVD mortality.

According to normal hyperuricemia and hypouricemia definitions, the prevalences of hyperuricemia and hypouricemia were 18.1 and 16.7%, respectively. Previous studies have indicated that sex affects SUA metabolism and the normal value range. Therefore, sex stratification analysis was carried out. Considering that FRS was a potential confounding factor, the Cox regression model was adjusted for FRS between males and females. The results revealed that the second quartile of SUA level (242.0–312.0 μmol/L) had the least mortality compared with other quartiles. In addition, RCSs and Kaplan–Meier survival estimation indicated high and low SUA to be undesirable.

Because we enrolled HAR patients, baseline characteristics indicated no significant difference in stroke, DM, MI history, PTCA history, CABG history, hyperlipidemia, or smoking status. Nonetheless, we found that the use of diuretics and low eGFR correlated substantially with SUA; after controlling for eGFR and diuretic use, HR estimates suggested only marginal changes, and the data only demonstrated some collinearity among eGFR, diuretics, and SUA. Consequently, the results revealed an independent association between SUA, all-cause mortality, and CVD mortality.

The relationship between SUA and mortality has been explored in previous studies. Similarly, several studies including a longitudinal Taiwanese cohort study (23), US adult cohort study (24), Korean study (25, 26) and Chinese chronic kidney disease study (17) reported U-shaped associations between SUA and all-cause mortality as well as cardiovascular mortality. Nevertheless, the different patient populations recruited might affect the occurrence of ending events to affect different SUA cutoff values and ranges of variation. Our study assessed values providing better prediction by X-Tile Software and ROC curves. After adjusting for confounding factors, this research revealed that the lowest mortality for a range of SUA of 180.5–370.5 μmol/L or 3.03–6.22 mg/dL (male) and 165.7–327.65 μmol/L or 2.78–5.50 mg/dL (female). Although early studies, including the Framingham Heart Study (27), Atherosclerosis Risk In Communities study (28), and Vorarlberg Health Monitoring and Promotion Program (29), reported a relationship between SUA and CHD and death, no U-curve distribution was observed because the studies mainly focused on patients with hyperuricemia. Recently, the *Journal of Hypertension, High Blood Pressure*, and *Cardiovascular Prevention* all published cutoff points (30, 31). Notably, the URRAH Project investigating hyperuricemia correlations with all-cause mortality and CVD mortality reported the best cutoff values of 4.7 and 5.6 mg/dL, respectively (31). Although our findings were similar, AUCs were smaller than the URRAH Project's results. The reason may be that ROC curves only assess statistical performance for single-trend variables, and the statistical efficiency is insufficient to demonstrate a U-shaped curve. For this reason, our study involved adding transformed SUA variables by RCS to a Cox regression model and calculating predictive all-cause and CVD-cause mortality scores. Then, the optimum predictive effect of the SUA level was determined using ROC analysis. This may explain why the AUC was slightly smaller but the HR higher than in the URRAH Project.

From the perspective of pathophysiology, previous studies have revealed that hyperuricemia may have detrimental effects on the endothelium and functions of platelets (32), induce oxidative stress and activate the local renin–angiotensin system in cultured vascular smooth muscle cells (33), lead to attenuated nitric oxide bioavailability, and promote the proliferation of vascular smooth muscle (32, 34, 35). Further studies have reported that reduced SUA levels may contribute to the treatment of hyperuricemia-associated diseases (36, 37).

Notably, hypouricemia was also found to be associated with all-cause mortality and cardiovascular mortality. Recently, several studies, including USA Adults Study, reported similar results (24). At present, there is no recognized standard for the diagnosis of hypouricemia. According to the definition of hypouricemia in most previous studies (16, 17, 38, 39), the reference limit was defined as a level of SUA < 149 μmol/L or 2.5 mg/dL. A possible etiology by which hypouricemia increases all-cause mortality may be caused by malignancy (40, 41), DM, and concomitant medication (42, 43). Nevertheless, these studies were limited by the absence of important covariates, such as smoking status and BMI. Recently, growing evidence suggests that SUA plays an important role in immune regulation and tumor inhibition (43). Therefore, the increase in mortality due to hypouricemia is partly owing to cancer incidence (44). Meanwhile, malignant tumors, as a kind of consumptive disease, have repeatedly appeared later in hypouricemia (45). These details may partly explain why hypouricemia patients tend to have higher all-cause mortality. SUA also acts as an antioxidant that can interact with hydrogen peroxide and hydroxy radicals to effectively scavenge free radicals in the body, thus protecting vascular endothelial cells (12). This research also indicates that patients with hypouricemia have increased all-cause and CVD mortality.

In addition to hyperuricemia and hypouricemia, age, smoking status, hypertension, ischemic stroke, MI, PAD, BMI, and lower eGFR were independent mortality risk factors in this study.

In conclusion, the findings reveal a U-shaped relationship between SUA level and all-cause mortality and cardiovascular mortality in HAR patients, regardless of sex. The results indicate that hyperuricemia and hypouricemia increased mortality and that SUA may serve as an easily tested index to predict mortality. Therefore, more intensive management regarding SUA is needed in clinical practice, especially in HAR patients. In addition, well-designed clinical trials focusing on SUA and its concomitant changes are needed to prevent all-cause and cardiovascular mortality.

### Study Limitations and Strengths

First, subjects with HAR were enrolled to assemble a study population as homogeneous as possible, as HAR patients have a poor prognosis. Thus, the results cannot be extended to the entire population. Second, as follow-up participants were contacted by annual phone interviews, our results may have information bias. In addition, some patients had poor compliance, and withdrawal bias may exist. Finally, the follow-up time was not long compared with Western country prospective cohort studies. Hence, the data from this research are not comprehensive, and additional studies are needed.

The strengths of our study include its prospective design and reliable assessment of mortality and cardiovascular events. Second, this research was a multicenter prospective cohort registration study with little heterogeneity. This cohort study also had a longer follow-up time and larger sample size than other Chinese studies.

## Conclusion

After adjusting for sex and other covariates, this study revealed that SUA level shows a U-shaped relationship with all-cause mortality and cardiovascular mortality. Abnormal SUA values correlated strongly, independently, and inversely with all-cause and cardiovascular mortality. Long-term average SUA levels >370.5 μmol/L or 6.22 mg/dL (male) and >327.65 μmol/L or 5.50 mg/dL significantly increased the risk of all-cause mortality and CVD mortality in females. Meanwhile, low-level SUA <180.5 μmol/L or 3.03 mg/dL (male) and <165.7 μmol/L or 2.78 mg/dL (female) was an independent risk factor for all-cause mortality and CVD mortality. Age- and sex-adjusted survival ROC curve analyses indicated that SUA level can predict all-cause mortality, with an AUC of 0.702 (95% CI = 0.692–0.725, *P* < 0.001), and CVD mortality, with an AUC of 0.711 (95% CI = 0.697–0.742, *P* < 0.001). After adjusting for confounding factors, compared with the SUA 180.5–370.5 μmol/L subgroup, HRs for all-cause mortality and cardiovascular mortality were 2.01 (95% CI = 1.35–2.56) and 2.08 (95% CI = 1.42–2.57) and 1.79 (95% CI = 1.45–2.57) and 2.09 (95% CI = 1.47–2.62), respectively, in males in SUA (0–180.5) and SUA >370.5 μmol/L subgroups and 1.98 (95% CI = 1.25–2.53) and 2.04 (95% CI = 1.39–2.61), and 1.89 (95% CI = 1.31–2.67) and 2.02 (95% CI = 1.43–2.74), respectively, in females in SUA (0–180.5) and SUA >370.5 μmol/L subgroups. In addition to hyperuricemia and hypouricemia, age, smoking status, hypertension, ischemic stroke, MI, PAD, BMI, and lower eGFR are independent mortality risk factors.

## Data Availability Statement

The original contributions presented in the study are included in the article/supplementary material, further inquiries can be directed to the corresponding author/s.

## Ethics Statement

The studies involving human participants were reviewed and approved by the ethics committee of Tongji University (NCT03616769). The patients/participants provided their written informed consent to participate in this study. Written informed consent was obtained from the individual(s) for the publication of any potentially identifiable images or data included in this article.

## Author Contributions

YC had full access to all of the data in the study and takes responsibility for the integrity of the data and the accuracy of the data analysis. YC: study concept and design, analysis and interpretation of data, drafting of the manuscript, critical revision of the manuscript for important intellectual content, and statistical analysis. YC and SX: acquisition of data. YC and YX: study supervision. All authors contributed to the article and approved the submitted version.

## Conflict of Interest

The authors declare that the research was conducted in the absence of any commercial or financial relationships that could be construed as a potential conflict of interest.

## Abbreviations

CVD: cardiovascular disease
SUA: serum uric acid
ABI: ankle–brachial index
HAR: high atherosclerotic risk
RCS: restricted cubic splines
BMI: body mass index
TC: total cholesterol
HDL-C: high-density lipoprotein cholesterol
LDL-C: low-density lipoprotein cholesterol
DM: diabetes mellitus
HR: hazard ratios
FRS: Framingham risk score
PAD: peripheral arterial disease
SBP: systolic blood pressure
DBP: diastolic blood pressure
IS: ischemic stroke
CRI: chronic renal insufficiency
DL: dyslipidemia
eGFR: estimated glomerular filtration rate
CHD: coronary heart disease
PCI: percutaneous coronary stent implantation
CABG: coronary artery bypass grafting
MI: myocardial infarction
Cr: serum creatinine
ROC: receiver operating characteristic
AUC: area under the curve.
